# Novel PRD-like homeodomain transcription factors and retrotransposon elements in early human development

**DOI:** 10.1038/ncomms9207

**Published:** 2015-09-11

**Authors:** Virpi Töhönen, Shintaro Katayama, Liselotte Vesterlund, Eeva-Mari Jouhilahti, Mona Sheikhi, Elo Madissoon, Giuditta Filippini-Cattaneo, Marisa Jaconi, Anna Johnsson, Thomas R. Bürglin, Sten Linnarsson, Outi Hovatta, Juha Kere

**Affiliations:** 1 Department of Biosciences and Nutrition, Karolinska Institutet, Novum, 141 83 Huddinge, Sweden; 2 Center for Innovative Medicine, Karolinska Institutet, Huddinge 141 83, Sweden; 3 Science for Life Laboratory, Tomtebodavägen 23 A, Solna 171 21, Sweden; 4 Department of Clinical Science, Intervention and Technology, Karolinska Institutet, Karolinska University Hospital Huddinge, Stockholm 141 86, Sweden; 5 ProCreaLab SA, Lugano CH-6900, Switzerland; 6 Department of Pathology and Immunology, Faculty of Medicine, Geneva University, 1 rue Michel-Servet, Geneva 4, 1211, Switzerland; 7 Department of Medical Biochemistry and Biophysics, Karolinska Institutet, Stockholm 171 77, Sweden; 8 Molecular Neurology Research Program, University of Helsinki and Folkhälsan Institute of Genetics, Biomedicum 1, Haartmaninkatu 8, Helsinki 00290, Finland

## Abstract

Transcriptional program that drives human preimplantation development is largely unknown. Here, by using single-cell RNA sequencing of 348 oocytes, zygotes and single blastomeres from 2- to 3-day-old embryos, we provide a detailed analysis of the human preimplantation transcriptome. By quantifying transcript far 5′-ends (TFEs), we include in our analysis transcripts that derive from alternative promoters. We show that 32 and 129 genes are transcribed during the transition from oocyte to four-cell stage and from four- to eight-cell stage, respectively. A number of identified transcripts originates from previously unannotated genes that include the PRD-like homeobox genes *ARGFX, CPHX1, CPHX2, DPRX, DUXA, DUXB* and *LEUTX*. Employing *de novo* promoter motif extraction on sequences surrounding TFEs, we identify significantly enriched gene regulatory motifs that often overlap with Alu elements. Our high-resolution analysis of the human transcriptome during preimplantation development may have important implications on future studies of human pluripotent stem cells and cell reprograming.

## Abstract (web summary)

Understanding human preimplantation development is invaluable for human reproduction and stem cell research. By employing single-cell RNA sequencing in oocytes, zygotes and single blastomeres, Töhönen *et al.* identify new regulatory factors and sequences that drive early human preimplantation development.

## Graphical abstract

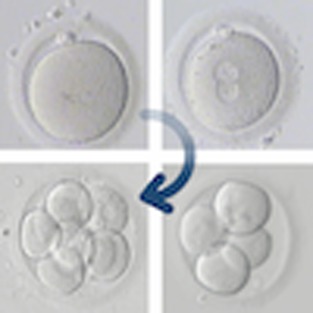

Human preimplantation development starts with the fusion of the egg and sperm pronuclei in the zygote and requires both embryonic genome activation (EGA) and degradation of maternal transcripts during the first 3 days after fertilization. Embryo compaction and lineage decision to either inner cell mass or trophectoderm occur thereafter before implantation into the uterus. The study of early human development has been based on a small number of samples, often pooled, due to the sparsity of material and methodological reasons, thus lacking single-cell resolution and transcriptome-wide approach and resulting in incomplete data^1^,^2^,^3^. We sought to overcome these limitations to obtain a detailed view of the first days of human preimplantation development based on the full annotation of messenger RNA (mRNA) start sites in single cells up to day 3, or three cell divisions after fertilization. The timing and success of the first cell divisions has been shown to be of crucial importance for successful blastocyst formation also in assisted reproduction^4^.

Our study differs from all previous in three essential ways. First, we analyse over 300 single human oocytes, zygotes, day 2 and day 3 blastomeres, increasing the number of cells over 10-fold compared with recent studies^5^,^6^. Second, we identify alternative promoters for genes using single-cell-tagged reverse transcription (STRT), a multiplex-tagged method for single-cell poly(A)-tailed RNA sequencing^7^ that detects the very 5′-end of every transcript, here called transcript far 5′-ends (TFEs; Supplementary Note 1). We quantify gene expression based on these transcription start sites. Third, using synthetic RNA spike-in normalization implemented computationally in SAMstrt^8^, we annotate expression in absolute rather than relative terms, allowing an improved resolution of transcriptional activity from cell cleavage effects and mRNA degradation. Importantly, in a situation where cell size is reduced by successive cell divisions, as in preimplantation development, the commonly used normalization methods may yield misleading interpretations. Our results suggest novel insights into the regulation of early human development and identify possible new factors for use in cell reprogramming, maintenance of pluripotency and induced pluripotent stem cell (iPS cell) biology.

## Results

### Single-cell sequencing of oocytes and cleavage stage embryos

**Figure 1:**
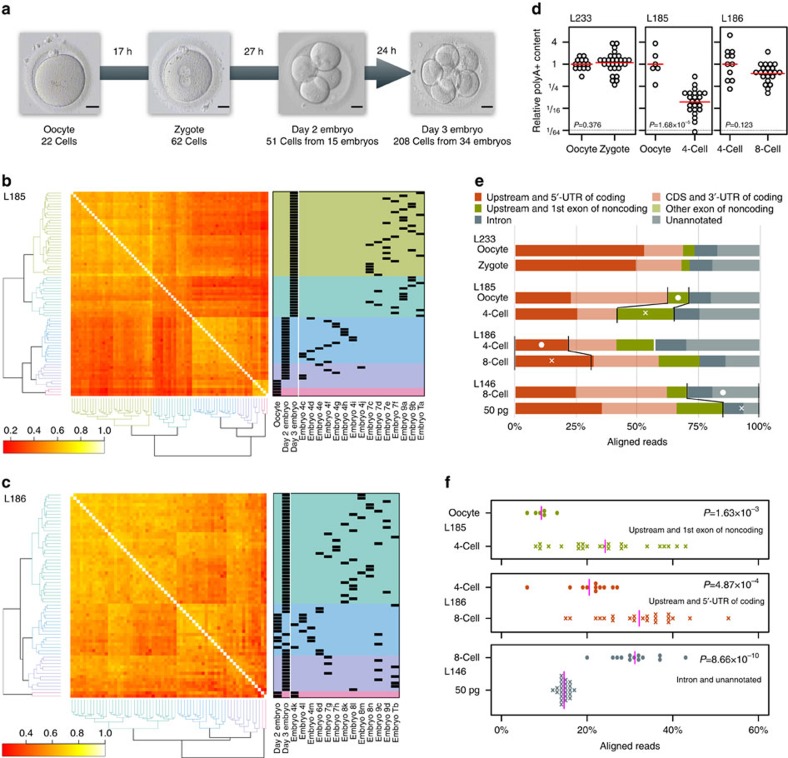
Overview of the study and changes in total cellular RNA content. (**a**) Microphotographs and number of cells used from each developmental stage studied. A total of 348 cells were collected at oocyte and zygote stage, day 2 (4–6 blastomeres) and day 3 (7–10 blastomeres) after fertilization. Scale bar, 20 μm. (**b**) Heterogeneity of gene expression among oocytes, day 2 and day 3 embryo blastomeres. The heatmap shows Spearman correlation coefficient (in complete pairs of observations) between the samples in library L185. The correlation matrix is sorted by hierarchical clustering with Euclidean distance and complete linkage method. (**c**) Spearman correlation coefficient between the samples in library L186 showing biological variation in day 2 and day 3 blastomeres. (**d**) Comparison of relative poly(A)-tailed RNA content between the developmental stages from plates L233, L185 including exactly staged 4-cell embryos (*n*=23), and L186 including exactly staged 4-cell (*n*=11) and 8-cell (*n*=22) embryos. Relative poly(A)-tailed RNA content per cell is calculated as the ratio of total mapped reads on genome references to mapped reads on spike-in references with centering by average of the ratios in earlier stage. Red line indicates average level, and *P* value is the assessment of the mean rank difference by Wilcoxon rank sum test. (**e**) A comparison of overall proportion of TFEs according to genome annotation. The comparisons are made between oocyte to zygote, oocyte to 4-cell, and 4- to 8-cell. The annotated groups marked with dots or crosses and separated by black lines are plotted separately in **f**. (**f**) Proportion of annotated reads for functional transcripts from groups ‘upstream and 5′UTR of coding', ‘upstream and 1st exon of noncoding', and ‘intron' and ‘unannotated' in the transitions from oocyte to 4-cell, 4- to 8-cell and comparison of 8-cell stage blastomeres to human brain RNA. Purple lines indicate average ratio, and *P* value is the assessment of the mean rank difference by Wilcoxon rank sum test. The variation between 8-cell stage blastomeres was more than pure technical variation represented by human brain samples, suggesting biological variation. 4-cell, four-cell; 8-cell, eight-cell.

We collected 348 single cells, oocytes, pronuclear zygotes (one-cell embryos) and isolated blastomeres from day 1 to day 3 embryos (two- to 10-cell stages) donated for research (Fig. 1a; Supplementary Table 1; Supplementary Movie 1). As controls for somatic expression profiles and technical variation, we prepared 24 replicas of 50 pg human brain total RNA. Assuming 5% mRNA content in total RNA, the brain sample mRNA input would be ∼2.5 pg, whereas a single oocyte may have an order of magnitude more mRNA^9^. Thus, in eight-cell stage embryos there would be ∼2.5 pg of mRNA per blastomere, which is in relatively good agreement with the effect of cell division and possible maternal RNA degradation. Therefore, the replicate brain RNA samples are valid as controls for estimating technical variation (no biological variation between the technical replicates).

In total, we sequenced 372 samples (348 embryo samples and 24 technical controls, Supplementary Data 1). The samples were processed as six STRT libraries, three of them specifically designed to address developmental stage comparisons: (i) library L233 to compare oocytes and zygotes; (ii) L185 to investigate the early wave of EGA by comparing oocytes and four-cell blastomeres; and (iii) L186 to study the four-to-eight-cell transition comprising the major EGA. To confirm the consistency with another RNA sequencing method and previous publications of human embryo development, we sequenced four single-zygote libraries using the Tang method^10^ and compared our results from single oocytes with previously published data^5^, shown in Supplementary Note 2.

### Assessment of technical and biological variation

We calculated Spearman correlations between the 14 oocytes on L233 using all pairs of observations. All combinations were significantly correlated (*P* value<0.05 with Bonferroni correction), and the mean coefficient was 0.7044. We also calculated Spearman correlations between the 23 replicates of 50 pg of human brain RNA on L146 (Supplementary Fig. 1); these samples served as a control to give an estimation of technical variation, whereas the oocytes also gave an estimation of biological variation. The 23 brain samples were significantly correlated (*P* value<0.05 with Bonferroni correction), with a mean coefficient 0.6612. The most likely interpretation of the lower correlation in the control brain samples compared with the oocyte samples is the ∼10-fold lower amount of input mRNA. The reduction of correlation coefficient by low amount of RNA is well known.

We performed clustering of the correlation coefficients, which means grouping of cells according to similar expression patterns (Fig. 1b,c). We observe an interesting pattern of biological variation between the day 3 blastomeres, suggesting either asynchrony of cell division, difference in the rate of maternal RNA degradation, first signs of differentiation, or a combination of all three. Our analysis does not allow a deeper dissection of the causes of the heterogeneity at this point. On the basis of our own observations and previous reports^5^,^6^, we performed all further analyses, except the definition of TFEs (Supplementary Note 1) and the Spearman correlation analyses, with exactly four-cell and eight-cell blastomeres. Future studies of human preimplantation embryos could combine continuous video recording of developmental timing with the recently published method of simultaneous single-cell sequencing of both transcriptomes and genomes^11^ to improve the resolution and identify any genomic or transcriptomic heterogeneity within single cells of multicellular embryos.

### Changes in total cellular RNA content

In total, 1.91 billion (1,909,944,216) sequenced STRT reads derived from 308 cells representing 131 oocytes or embryos and 23 human brain RNA samples were analysed (Supplementary Table 1a–d). After quality control and exclusion of failed samples, we used 1.58 billion (1,582,567,706) mapped STRT reads (82.9% genomic mapping rate) in analyses. Importantly, STRT uses poly(T) priming for complementary DNA (cDNA) synthesis and all our data refer to poly(A)-tailed RNA content. The counting of poly(A)-tailed RNA molecules in oocytes, zygotes, four- and eight-cell embryo blastomeres reveals unchanged poly(A)-tailed RNA content in oocytes and zygotes, but significant reduction of cellular poly(A)-tailed RNA content in four-cell blastomeres, consistent with previous data and the reduction in cell size after two cell divisions^9^,^12^,^13^ (Fig. 1a,d). We observe a further slight reduction between four- and eight-cell blastomeres, consistent with the twofold reduction in cell size. Therefore, the use of synthetic spike-in RNA molecules for normalization, as implemented in SAMstrt, is critical to correctly assess transcriptome changes in the comparison of cells with very different amount of poly(A)-tailed RNA (Supplementary Fig. 2).

### Identification of novel transcription start sites

We classify TFEs according to their annotated genomic positions. Overall, the proportions of genomic locations of mapped STRT reads are similar between oocytes and zygotes (Fig. 1e). Comparing oocytes and four-cell blastomeres, the significant reduction of transcripts affects proportionately more coding genes than transcripts corresponding to noncoding genes, consistent with the degradation of maternal coding transcripts (Fig. 1e,f). The four-to-eight-cell transition is characterized by a significant increase in coding transcripts, while noncoding transcripts are increased in the oocyte-to-four-cell transition (Fig. 1e,f). At all stages, the proportion of intronic or unannotated TFEs is higher than in the human adult brain samples, suggesting transcription from alternative promoters of defined genes, or completely novel genes, during preimplantation development.

### Single-cell gene expression during EGA

**Figure 2:**
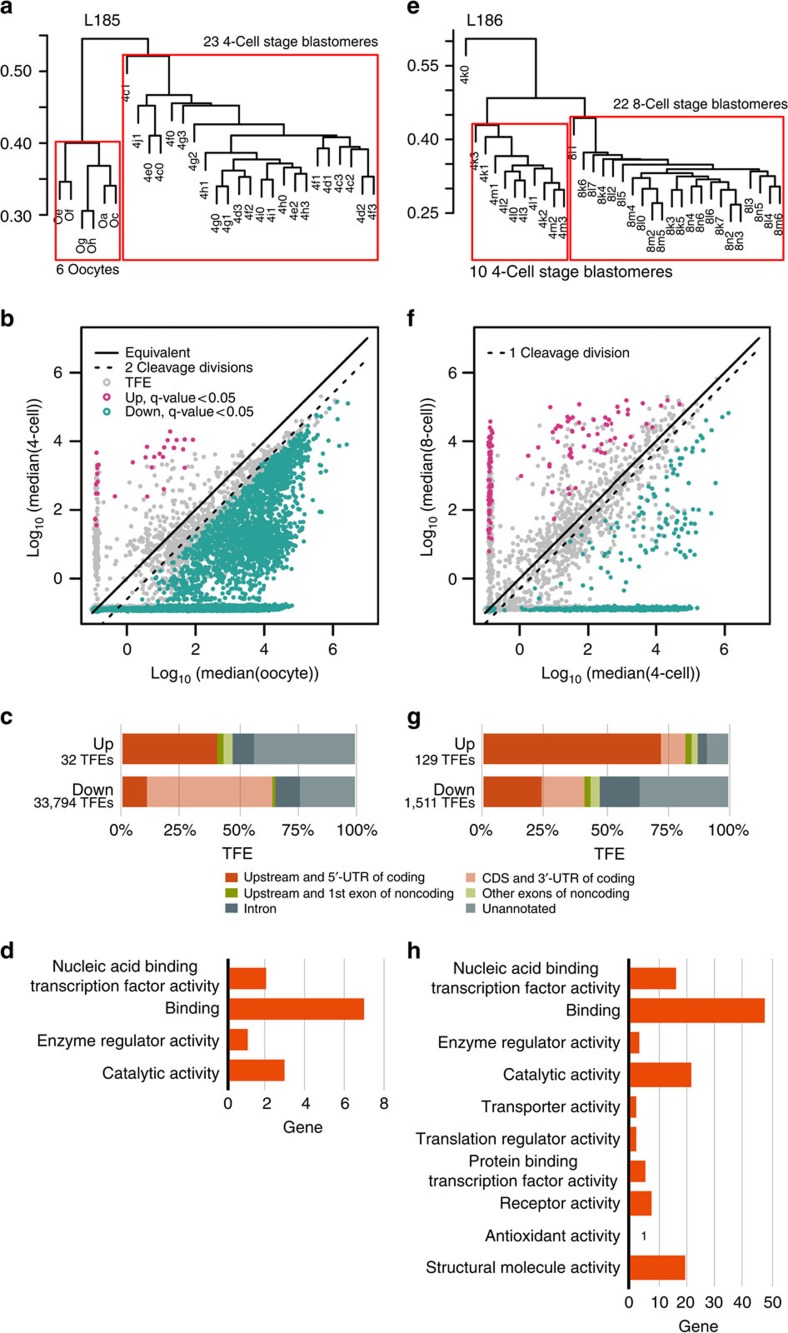
Analysis of single-cell gene expression levels during EGA. To exclude library bias effect, we performed the comparisons between cells within the same STRT libraries. (**a**) Analysis of early EGA (oocyte-to-4-cell transition). Pvclust hierarchical clustering of the cells on plate L185 shows that cells from different cell stages are clearly isolated from each other, whereas cells from the same cell stage cluster together. The cells within the red boxes were included in downstream analyses. (**b**) Upregulated (red dots), downregulated (green dots) and genes showing no significant change (grey dots) in the two stages. The dotted line marks the cell division effect on cellular RNA content (two cell divisions from oocyte to 4-cell stage and one cell division from 4-cell to 8-cell stage). (**c**) A comparison of overall proportions of TFEs according to genome annotation for significantly upregulated and downregulated TFEs during early EGA. The 32 upregulated TFEs in the early EGA included known start sites for coding genes (red bar), but also unannotated transcription sites (light grey). Downregulated TFEs, in contrast, consisted of >50% internal exons or 3′UTR of coding genes (pink), consistent with partially degraded transcripts. (**d**) GO molecular function classification of the 32 upregulated coding genes during early EGA. (**e**) Analysis of major EGA (4-to-8-cell transition). Pvclust hierarchical clustering of the cells on plate L186. The cells within the red boxes were included in downstream analyses, while the outlier cell was excluded from further analyses. (**f**) Upregulated (red dots) and downregulated (green dots) and genes showing no significant change (grey dots). The dotted line marks the cell division effect on cellular RNA content as described above. (**g**) A comparison of overall proportions of TFEs according to genome annotation for significantly upregulated and downregulated TFEs during EGA. More than 70% upregulated TFEs mapped to annotated coding gene start sites, compared to <25% of the downregulated transcripts. (**h**) GO molecular function classification of the 129 upregulated coding genes during major EGA. 4-cell, four-cell; 8-cell, eight-cell.

To analyse the up- and downregulated genes in the two transitions corresponding to early and major EGA, we compared the oocyte-to-four-cell and four-to-eight-cell transitions, respectively. We analysed only embryos with exactly four or eight cells to avoid variation arising from inexact timing. The early EGA at oocyte-to-four-cell transition (Fig. 2a–d) is characterized by a significant upregulation of 32 TFEs (Supplementary Data 2) and a massive reduction of total TFEs (Supplementary Data 3) consistent with active maternal transcript degradation^9^,^12^,^13^ and passive reduction (mRNA content division between daughter blastomeres). The analysis of the 32 upregulated TFEs show that 12 map to the 5′-untranslated region (UTR) of coding genes (*ARHGAP28, DUXA, H2AFZ, KHDC1L, PRAMEF1, RBBP6, SHC4, SRSF, TRIM48, USP29, ZSCAN4* and *LOC440563*), one to an intron (*LEUTX*), one maps upstream of a coding gene (*LOC649330*), and, surprisingly, the rest map to unannotated genomic sites (Fig. 2c). In contrast, intragenic exons or 3′UTR sequences of coding genes are strongly over-represented among downregulated TFEs, suggesting partially degraded transcripts resulting in part from 5′-exonuclease activity^14^.

The analysis of four-to-eight-cell transition (Fig. 2e–h), that is, the major EGA, reveals 129 significantly upregulated TFEs (Supplementary Data 4) of which nearly 75% correspond to the 5′-exons of coding genes (Fig. 2g) possessing mostly catalytic and binding activities (Fig. 2h). The profile of the downregulated transcripts (Supplementary Data 5), again, corresponds to unannotated, intronic and middle or 3′-exonic transcript sequences (Fig. 2g), consistent with partially degraded transcripts.

Comparing with the previous report of 2,495 significantly upregulated genes between 4- and eight-cell stage by Yan *et al.*^5^, the much smaller number of genes upregulated in our study may seem surprising. The obvious reason for the large differences in reported gene numbers is the method used for data normalization and interpretation. The commonly used mean normalization works poorly in situations where there is huge imbalance between the reduction and gain of transcripts, as is the case in the day 2 and day 3 blastomeres (Supplementary Fig. 2). Thus, quantification using synthetic spike-in RNA molecules for normalization provides an unambiguous method in this unbalanced situation and allows for a more accurate interpretation of the data^8^. Many biological processes work as cascades depending on only a few key genes, one example being the four Yamanaka factors needed to achieve reprogramming of differentiated cells to a pluripotent stage^15^,^16^. Our implication of 32 and 129 genes, respectively, in the consecutive days of EGA may indeed reflect EGA more accurately than previous studies and these genes may suffice to initiate the embryo development.

### Identification of *de novo* regulatory motifs within Alu elements

**Figure 3:**
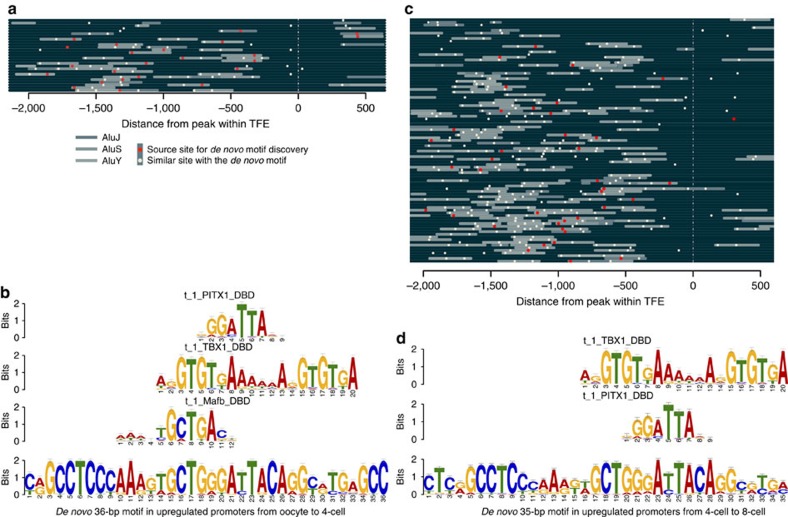
*De novo* DNA motif discovery in promoter of upregulated TFEs. (**a**) Conserved motifs in the promoter regions (at −2,000∼+500-bp distance of TFE cluster peak) present in 27 of the 32 TFEs upregulated in the transition from oocyte to 4-cell embryo. The red dots indicate the positions of the *de novo* 36-bp motifs, while the white dots indicate the positions of similar sequence to the *de novo* 36-bp motif. There are 50 Alus in the 27 *de novo* motif containing promoters in the oocyte to 4-cell transition (grey bars). (**b**) Known TF-binding sequences for PRD-like homeobox, T-box and bZIP and the first predicted *de novo* 36-bp DNA motif that includes these elements. Error bars indicate the confidence of a motif based on the number of sites. (**c**) Conserved motifs present in the promoters of 91 out of 129 TFEs upregulated at the major EGA. The promoters of upregulated TFEs show significant enrichment of Alu elements, with 208 Alus (grey bars) in the 91 *de novo* motif containing promoters. (**d**) T-box and PRD-like homeobox elements show similarities to a *de novo* 35-bp DNA motif sequence highly similar to the 36-bp motif and present in 39 promoters of the upregulated TFEs in the major EGA. Error bars indicate the confidence of a motif based on the number of sites.

We extracted sequence motifs in the genomic DNA around the upregulated TFEs to identify potential regulatory elements involved in EGA. If any sequence-specific DNA-binding protein is a candidate for either activating early transcription of nearby genes in *cis* or for preventing their leaky transcription before fertilization, such specific DNA motifs should be over-represented around transcriptional start sites (TSSs) of genes involved in EGA. We identify four significant *de novo* motifs at genomic positions −2,000 to +500 bp of the 32 upregulated TFEs in the early EGA (Supplementary Fig. 3). A 36-bp long *de novo* motif is identified within Alu elements in 23 of the 32 promoters at the four-cell stage including 5′UTR of *H2AFZ*, *KHDC1L* and *ZSCAN4* (Fig. 3a,b). The *de novo* motif overlapping with the Alu elements is similar to known consensus sequences of binding sites for bZIP, T-box and PRD-like homeodomain containing transcription factors (TFs; Fig. 3b, and the motif based on database analysis in ref. 17 and Supplementary Data 3). Furthermore, we find a highly similar motif, also with frequent Alu element overlap, in altogether 27 of the 32 TFE promoters (Fig. 3a; Supplementary Fig. 3).

Similar to the early EGA, we identify 13 significant *de novo* motifs (Supplementary Fig. 4) in the promoter regions of the 129 upregulated TFEs at eight-cell stage (Fig. 3c; Supplementary Data 4). We identify a 35-bp motif in 39 promoters of the TFEs (Fig. 3d; Supplementary Data 4). Similar sequences are spread over altogether 91 out of the 129 promoters, again with frequent overlap to Alu elements, especially AluY and AluS^18^ (Fig. 3c). The 35-bp motif is also similar to the known motifs for T-box and PRD-like homeodomain TFs (Fig. 3d).

Alu retrotransposon elements are the major short interspersed nucleotide element family present in the primate genome^18^, showing non-random distribution in the human genome with enrichment to GC-rich regions^19^. Interestingly, our data show an over-representation of Alu elements around 1–2 kb from the TSS of the upregulated TFEs. This is in accordance with Polak *et al.*^20^ who suggested a role for Alu elements in transcriptional regulation on the basis of the enrichment of Alu elements 2–5-kb upstream of TSSs and containing many TF-binding sites. Here we observe a frequency of about two Alu elements within 2 kb from the TSSs of developmental genes, that is, more frequently than the expected frequency of up to 1.1 (ref. 20).

### PRD-like homeobox genes regulate EGA

Homeodomain proteins function as DNA-binding TFs primarily in development and differentiation. The PRD-like homeobox genes encode homeodomains that are similar to the PRD class homeodomains, but they lack the actual PRD domain^21^. We find that *ZSCAN4*, a known inducer of iPS cells^22^, is highly expressed already at four-cell stage, and further accumulated at eight-cell stage. The TFE peak is within the second exon of *ZSCAN4* (Supplementary Fig. 5a). The promoter region around the TFE peak contains six sequences similar to the 36-bp *de novo* motif, all overlapping with three Alu elements on the same strand (Supplementary Fig. 5b). To investigate the activity of the putative promoters containing the predicted 36-bp *de novo* motif, we cloned a fragment of the putative *ZSCAN4* promoter up to 1,605-bp upstream of the TFE peak (Supplementary Fig. 5b) and placed it upstream of the luciferase reporter gene. The cloned promoter fragment increases luciferase expression fivefold, and co-transfection using the PRD-like homeodomain containing TFs *DUXA* or/and *OTX2* yields an up to 25-fold increase in luciferase expression (Supplementary Fig. 5c). Thus, the predicted novel *ZSCAN4* promoter containing the 36-bp motifs can drive the expression of downstream genes, and may be activated during the EGA.

### PRD-like homeobox genes in early development

**Figure 4:**
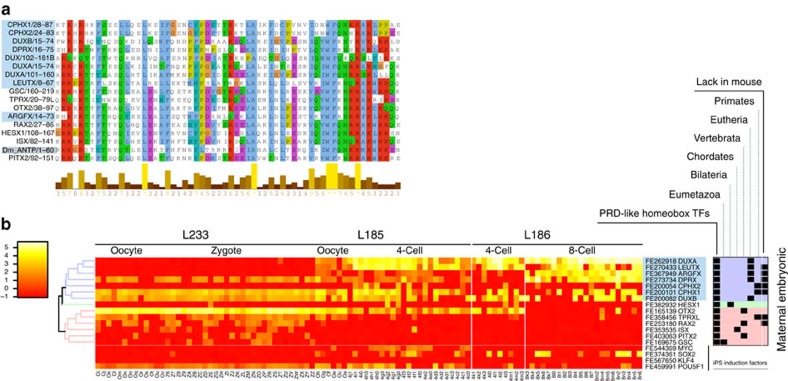
PRD-like homeobox genes expression during EGA. (**a**) Comparison of conserved homeodomains present in novel PRD-like homeobox genes cloned from early human development (blue shading) and in previously annotated genes (no shading). The highest degree of conservation can be found for the amino-acid residues responsible for forming the tertiary structure of the homeodomain. *Drosophila melanogaster* ANTP (grey shading) is given as reference for homeodomain proteins. The amino-acid sequences are aligned using ClustalW. (**b**) Early developmental expression pattern of the novel PRD-like homeobox genes (blue shading) and previously annotated genes. The expression profiles of the so-called Yamanaka factors (iPS induction factors) *MYC, SOX2, KLF4* and *POU5F1/OCT4* are shown for comparison. Heterogeneous expression between individual blastomeres at 8-cell stage (day 3 embryo) is seen for *LEUTX* and *CPHX1* expression in particular. In addition, a reciprocal patterning of *CPHX1* and *CPHX2* expression is detected in developmental stages up until 8-cell stage, with *CPHX1* being present in the oocyte, whereas *CPHX2* is upregulated in a few cells of 4-and 8-cell stage embryos. The box at the far right describes the presence (indicated by black squares) of the novel PRD-like homeobox genes and the previously annotated genes in the different evolutionary branches. Background colour in the box visualizes the two main clusters from the heatmap (clusters shown at far left).

Since the PRD-like homeodomain TF-binding motif is over-represented with high significance in both early and late waves of EGA (Supplementary Data 2 and 4), and the motif can activate transcription, we investigated further the expression of known PRD-like homeobox genes in human preimplantation development. There are 18 PRD-like homeobox genes that are significantly expressed during the early development (Supplementary Note 3). The detected TFEs for 14 of the 18 genes are compatible with functional transcripts, that is, (i) the TFE is at the 5′UTR; (ii) the transcript contains a complete open reading frame (ORF); and (iii) the ORF contains a complete homeodomain (Supplementary Data 6), as assessed by similarity to GenBank mRNAs and by cloning from independent whole-embryo libraries. Eight of the genes have unknown binding motifs^17^, but the amino-acid sequences of their DNA-binding homeodomains are similar to each other (Fig. 4a). Therefore, we propose that these 14 TFs are candidate binding factors to the *de novo* motif involved in EGA. The expression profiles of the 14 TFs represent three different groups of genes (Fig. 4b): maternal factors (*GSC, HESX1, ISX, NOBOX*, *OTX2*, *PITX2, RAX2* and *TPRXL*), embryonically activated (*ARGFX, DPRX, DUXA* and *LEUX*) and mixed (*CPHX1, CPHX2* and *DUXB*). The maternal factors tend to be conserved in many species, while the embryonic genes tend to have more species-specific expression. The conserved 14 TFs can also be grouped on the basis of evolutionary conservation as being present already in Eumetazoa (*GSC*), Bilateria (*HESX1*), Chordates (*ISX*), Vertebrates (*OTX2, PITX2* and *RAX2)*, Eutherians (*ARGFX, DPRX, DUXA, DUXB* and *LEUTX)* and Primates only (*CPHX2, CPHX1* and *TPRXL*). Several of the TFs lack orthologues in mouse (*ARGFX, CPHX1, CPHX2, DPRX, LEUTX, RAX2* and *TPRXL*) and many of them are activated in the zygotes or the later stages, suggesting novel primate-specific functions in early development^23^.

### PRD-like homeobox genes—novel regulators of EGA

Because many of these TFs (Supplementary Data 6) are poorly annotated in the public databases as incompletely predicted or completely lacking any cDNA evidence, we verified the start sites and full sequence of the transcripts by cloning and sequencing the cDNAs for seven of the novel TFs (*ARGFX, CPHX1, CPHX2, DPRX, DUXA, DUXB* and *LEUTX)* from single eight-cell stage embryo libraries. We find that the seven predicted TFs identified through novel promoters and currently lacking Genbank information produce full length ORFs with complete homeodomains (Fig. 4a; Supplementary Data 6; Supplementary Figs 6–10).

Our results suggest a new concept where retrotransposed elements can be regulatory elements for the transcription of genes functioning in EGA, supporting earlier reports about the importance of transcribed retroelements^24^ or suppression by retroelement regulation^25^ in preimplantation development. The transposition of the elements might provide new, sometimes species-specific, regulatory networks during evolution. Moreover, the previously unannotated TFs that we identify appear before embryo compaction and inner cell mass formation, suggesting novel regulation of early development and introducing possible new factors for use in blastomere reprogramming, maintenance of pluripotency and iPS cell biology.

## Methods

### Ethical statement

This study was reviewed and approved by the ethics review boards according to the applicable laws in Sweden and in Switzerland. All cells were donated by couples who underwent infertility treatment by *in vitro* fertilization. Cryopreserved cells not needed for treatment were donated by informed consent; the donated cells would otherwise be destined for destruction, because the legal storage time had been reached. Zygotes were collected, put in individual wells on a 96-well plate and lysed according to the protocol^7^ in Switzerland (authorization CE2161 of the Ticino ethical committee, Switzerland) before transfer to Sweden for sequencing. Oocytes and embryos were collected in Sweden (Dnr 2010/937–31/4 of the Regional Ethics Board in Stockholm).

### Human oocyte, zygote and blastomere collection

Non-fertilized MII oocytes were collected and the zona pellucidas were removed using acid Tyrode's solution (5 mg ml^−1^, Sigma-Aldrich). A small number of the unfertilized oocytes divided spontaneously during day 1 to two-cell stage. Zygotes were frozen at the pronuclear stage (2PN) 18–20 h after fertilization. After thawing, the zona pellucidas were removed. The dezoned cells were put into STRT lysis buffer in individual wells on a 96-well plate and thereafter immediately frozen on dry ice.

Cleavage stage embryos were frozen at four-cell stage on day 2 after fertilization. After thawing, embryos were allowed to develop until 6–10-cell stage in a sequential culture system (G1/CCM medium, Vitrolife) at 37 °C and 5% CO_2_, 5% O_2_. All blastomeres from each embryo were individually obtained by laser-assisted biopsy. Briefly, the embryos were held with a holding pipette and ∼50-μm holes were made in the zona pellucida using laser pulses. Individual blastomeres were aspirated through these holes and put one by one into STRT lysis buffer and frozen for downstream applications. All embryos used for blastomere biopsy were of equal size with little or no cytoplasmic fragmentation.

### Single-cell RNA sequencing using the STRT method

We applied STRT^7^, a highly multiplexed method for single-cell RNA sequencing that allows molecule counting. We analysed altogether 348 cells, including oocytes, zygotes and single blastomeres from 4- to 10-cell embryos (Supplementary Table 1). The sample and library preparation and sequencing were performed according to a published protocol^26^. Briefly, each well of the 96-well plate contained one cell in 5 μl of lysis buffer, a universal primer and a different template-switching helper oligo with a well-specific 6-bp sequence (Supplementary Data 1) enabling the cell-specific identification of sequencing reads. Reverse transcription reagents and eight synthetic spike-in RNAs^26^ (ArrayControl RNA spikes Ambion, cat. no. AM1780) were added to generate first-strand cDNA. After cDNA synthesis, the 96 individual samples were pooled into a single reaction and amplified by single-primer PCR using the universal primer sequence. The amplified samples were sequenced on the Illumina platform.

### Data pre-processing, alignments and quality control

STRT reads from oocytes, zygotes, four- and eight-cell stage blastomeres and 50 pg human brain RNA samples (Firstchoice human brain reference total RNA; Ambion, cat. no. 6,051) were first filtered, demultiplexed and trimmed by removing the 6-bp well-specific barcodes. Samples were excluded from further steps in case of shallow sequencing depth (<100-k reads per well) or no detected spike-in reads. Ratio of transcripts per cell was estimated by total reads per total spike-in RNA associated reads (all with sample-specific barcodes). After pre-processing, the reads were aligned to human UCSC genome hg19, ArrayControl RNA spikes and human ribosomal DNA complete repeat unit (GenBank: U13369) by TopHat version 2.0.6 (ref. 27; with options—library-type fr-secondstrand—min-anchor 5—coverage-search—bowtie1 and UCSC Gene as the transcriptome index), and annotated by genomic features that consider gene models and repetitive elements. Samples with either shallow sequenced reads (<5.34, which is mean−2 s.d. of log10 sequenced reads), low mapping rates (<60.65%, which is mean−2 s.d. of mapping rates) or shallow spike-in reads (<50 reads; 3,540 reads in average) were discarded from further analyses.

### Definition of transcript far 5′-ends

The aligned STRT reads were assembled by sample types using Cufflinks^27^ (with options --min-frags-per-transfrag 5—library-type fr-secondstrand) and counted as TFEs (Supplementary Note 1). In brief, the 5′-end regions of each transcript for each sample type represent a specific 5′-end of a poly(A)+ RNA in that particular sample. In any sample, TFEs from several RNA molecules typically overlap and represent sample specific first exons that we assign a unique ID (‘FE' followed by number). Overlapping TFE reads were counted by sample and normalized by the spike-in molecules. Also, the TFEs were compared with human genome annotation by UCSC Gene (6 February 2012). The TFEs were aligned to the genome and classified as the following: (i) overlapping with 5′UTR of coding transcript; (ii) upstream (up to 500 bp) of coding transcript; (iii) overlapping with coding sequence (CDS) of coding transcript; (iv) overlapping with 3′UTR of coding transcript; (v) overlapping with the first exon of noncoding transcript; (vi) upstream of noncoding transcript; (vii) overlapping with another exon of noncoding transcript; (viii) overlapping with an intron of any transcript; or (ix) unannotated position. The method of TFE-based quantification and the annotation are implemented as open-source software ( https://github.com/shka/STRTprep with scripts and the documents openly accessible; manuscript in preparation).

### Statistical test for differential expression

Before the differential expression tests, we applied pvclust^28^ to exclude outlier samples based on their expression profiles. After exclusion, we tested differential expression by SAMstrt^8^, which is SAMseq^29^ modified for spike-in-based normalization.

The downregulated TFEs are a mixture of passively reduced transcripts (due to cell volume reduction by cleavage) and actively reduced transcripts (by maternal transcript degradation). To eliminate the passive reduction effect by cell cleavages in statistical tests for differential expression between two stages, we correct for the number of cell divisions (four for comparison from oocyte to four-cell stage, two for comparison from four- to eight-cell stage) before SAMstrt and identify actively reduced TFEs as significantly reduced after the correction. No such correction was used to identify upregulated TFEs.

### Statistical test for correlation analysis

For clustering of samples with correlation coefficient matrix (Fig. 1b,c)), (i) zero-read expressions in the normalized expression profile were masked, (ii) Spearman correlation coefficients for all pairs of the (partly masked) normalized expression values were calculated and (iii) the coefficient matrix was clustered by complete linkage and Euclidean distance. For clustering of TFEs, or of chromosome arms, with the normalized expression profile, zero-read expressions were not masked.

### Promoter sequence analysis

We applied MEME^30^ (package version 4.9.0) for motif analysis within the active promoters. The promoters in our analysis are strand-specific sequences from 2,000-bp upstream to 500-bp downstream of the peak position within the clustered TFEs, and we assume that each promoter sequence may contain at most one occurrence of each motif. The significant motifs (E value<1.0 × 10^−200^ by MEME; red dots in Fig. 3a,b) were aligned to the promoters again by MAST^31^ (in the MEME package) to reveal actual motif distributions (*P* value<1.0 × 10^−10^; shown as white dots in Fig. 3a,c). The location of Alu elements within the promoters was based on Repeating Elements track by RepeatMasker in UCSC Genome Browser. The motifs were also compared with known motifs^17^ by TomTom^32^.

### Validation of novel transcripts by mRNA sequencing

To test the validity of our findings, we performed sequencing on single-cell mRNAs using the alternative method described by Tang *et al*^10^. Zygotes were frozen at the pronuclear stage (2PN) 18–20 h after fertilization. After thawing, the zona pellucidas were removed using Tyrode's solution followed by three washes in PBS–bovine serum albumin (1 mg ml^−1^) droplets. Each single cell was put into a 0.5-ml tube containing 4.45 μl of freshly prepared cell lysis buffer and subsequently treated according to the protocol by Tang *et al*^10^. In total, 12 single-zygote libraries were prepared, out of which four were sequenced on the SOLiD platform version 4 (Applied Biosystems). Cleavage stage embryos were frozen at four-cell stage on day 2 and at eight-cell stage on day 3 after fertilization. After thawing, the four- and eight-cell embryos were dezoned as previously described, followed by three washes in PBS–bovine serum albumin (1 mg ml^−1^) and put into lysis buffer^10^ for downstream analysis. A total of four eight-cell embryos were collected and prepared for sequencing. The library preparations were stopped before the fragmentation step to use these libraries for transcript cloning purposes.

### cDNA cloning of novel PRD-like homeobox gene transcripts

Single human eight-cell embryo cDNA libraries were used to clone the PRD-like homeobox TF gene transcripts. The libraries were initially prepared for sequencing according to the method described by Tang *et al.*^10^, but stopped before the fragmentation step and stored at −80 °C. *ARGFX*, *DPRX* and *DUXA* were cloned by PCR amplification using primers designed based on human RefSeq NM_001012729.1 (NCBI GeneID 503835), NM_001012728.1 (GeneID 503834) and NM_001012659.1 (GeneID 503582), respectively. The sequences containing ORFs for *CPHX1*, *CPHX2* and *DUXB* were predicted based on the TFEs FE200101, FE200082 and FE200054, respectively. The primer design for *LEUTX* was based on FE270433 located in an intron of the human RefSeq sequence, NM_001143832.1 (GeneID 342900), connected to the 3′UTR of predicted sequence. All primer sequences were designed by Primer-BLAST^33^ and are given in Supplementary Data 6.

The transcripts were amplified using specific primers (Supplementary Data 6) and Phusion High-Fidelity DNA polymerase (New England Biolabs) according to the manufacturer's instructions. *ARGFX*, *CPHX1* and *CPHX2* were amplified using a touchdown PCR program: 98 °C for 30 s; 24 cycles of 98 °C for 10 s, annealing for 30 s, temperature decreasing from 63 °C to 56 °C, 1 °C/3 cycles, 72 °C for 30 s; 16 cycles of 98 °C for 10 s, 55 °C for 30 s, 72 °C for 30 s; final extension 72 °C for 10 min. To amplify *DUXA*, *DPRX* and *LEUTX*, the following program was used: 98 °C for 30 s; 40 cycles of 98 °C for 10 s, 65.9 °C (*DUXB*)/67.9 °C (*DPRX*)/71.5 °C (*DUXA* and *LEUTX*) for 30 s, 72 °C for 1 min; final extension 72 °C for 10 min. The PCR products were cloned into pCR4Blunt-TOPO vector using Zero Blunt TOPO PCR Cloning kit (Invitrogen) and the inserts were verified by Sanger sequencing (Eurofins Genomics).

### Construction of *DUXA* and *OTX2* expression vectors

To overexpress *DUXA* and *OTX2* in mammalian cells, the cDNAs were cloned into a modified pFastBac expression vector. The modified pFastBac vector called CMVe.EF1α.eGFP-WPRE (kindly provided by Professor Shu Wang, Institute of Bioengineering and Nanotechnology, Singapore^34^) was further modified as follows: the eGFP and WPRE elements were removed from the vector by EcoRI and XbaI restriction digestion followed by blunting by T4 DNA Polymerase (Thermo Scientific) and dephoshorylation by Antarctic Phosphatase (New England Biolabs). The IRES, eGFP and WPRE insert was amplified by Phusion DNA Polymerase (New England Biolabs) from the vector FSynIGW (Addgene) with the introduction of the AscI and PacI restriction sites using the following primers: BamHI_AscI_PacI_IRES_Fwd 5′-tACCGGTGGATCCGGCGCGCCtaTTAATTAAgatccgcccctctccctccc-3′ and WPRE_Rev 5′-CTCGAGGTCGACGGTATCGAT-3′. The insert was phosphorylated by T4 Polynucleotide kinase (New England Biolabs) and ligated into the vector with T4 DNA Ligase (New England Biolabs). The strand confirmation was performed by digesting the vector with BamHI, which resulted in a single band for the correct orientation and double band for the opposite orientation. The final structure of the expression cassette is 5′-CMVe-EF1α-AscI-PacI-IRES-eGFP-WPRE-3′ in the pFastBac vector.

### Construction of novel *ZSCAN4* promoter reporter vector

The predicted *ZSCAN4* promoter region containing several sequences similar to our predicted 36 bp *de novo* DNA-binding motif was PCR amplified and cloned into pGL4.11 and pGL4.25 luciferase vectors (Promega), the former being a basic vector and the latter containing a minimal promoter. The promoter region was amplified with a primer pair giving a promoter length of 1,605 bp (chr19:58180248–58181870) containing a TATA-box sequence at the very 3′-end of the amplified fragment. The primer pair was as follows: 2 forward 5′-ATGGTACCCCTGGAATTGGCACAGGAGT-3′ and 2 reverse 5′-ATTGCTAGCTGATGTGCCTCCTAAGGCTG-3′. The primers contained either KpnI or Nhe1 restriction site at their 5′-end.

### Luciferase reporter assay

The HEK-293 cells (ATCC, Middlesex, UK) were seeded on 48-well plates in Dulbecco's modified Eagle medium containing 1 g l^−1^ glucose, L-glutamine and pyruvate and supplemented with 10% FBS and 2 mM L-glutamine (all from Gibco). Cells were grown overnight at 37 °C in 5% CO_2_ and subsequently transfected with different combinations of luciferase vector constructs, pFastBac vector constructs and Renilla luciferase vector pGL4.74 (Promega). The concentrations of single constructs were as follows: Luciferase vector 100 ng per well, pFastBac vector 100 ng per well and Renilla luciferase vector 10 ng per well. The transfections were performed using Lipofectamine 2000 (Invitrogen) 0.5 μl per well according to the manufacturer's instructions. Cells were incubated at 37 °C in 5% CO_2_, harvested 24 h after transfection and subjected to Dual luciferase assay (Promega) according to the manufacturer's protocol. Luciferase signals were measured using a TECAN infinite M200 (Tecan).

## Additional information

**Accession codes:** RNA-Seq data have been deposited in European Nucleotide Archive under accession code PRJEB8994.

**How to cite this article:** Töhönen, V. *et al.* Novel PRD-like homeodomain transcription factors and retrotransposon elements in early human development. *Nat. Commun.* 6:8207 doi: 10.1038/ncomms9207 (2015).

## Supplementary Material

Supplementary Information Supplementary Figures 1-10, Supplementary Table 1, Supplementary Notes 1-3 and Supplementary References

Supplementary Data 1 Detailed sample information and library layout

Supplementary Data 2 Significantly accumulated TFEs in transition from oocyte to 4-cell

Supplementary Data 3 Significantly decreased TFEs in transition from oocyte to 4-cell

Supplementary Data 4 Significantly accumulated TFEs in transition from 4-cell to 8-cell

Supplementary Data 5 Significantly decreased TFEs in transition from 4-cell to 8-cell

Supplementary Data 6 PRD-like homeobox transcription factors in human preimplantation development

Supplementary Movie Individual blastomere isolation method. This movie is a demonstration of how individual blastomeres were isolated from embryos for STRT sequencing. The movie shows a representative day 3 embryo (8-cell stage) kept in place with a holding pipette. Laser pulses were applied to degrade zone pellucida; the diameter of the target area is approximately 40 μm. The movie subsequently shows change of optics for a wider-angle image and aspiration of individual blastomeres from the embryo. All eight blastomeres were recovered intact and placed in the culture medium. Not shown in the movie is the transfer of single cells into plate wells containing STRT lysis buffer.
