# Supplementary material for: Novel PRD-like homeodomain transcription factors and retrotransposon elements in early human development
Source: Nat Commun. 2015 Sep 11;6:8207. doi: 10.1038/ncomms9207 (PMC4569847; doi:10.1038/ncomms9207)
Supplement: Supplementary Information — Supplementary Figures 1-10, Supplementary Table 1, Supplementary Notes 1-3 and Supplementary References [file ncomms9207-s1.pdf]

Novel PRD-like homeodomain transcription factors and retrotransposon elements in early human development

Virpi Töhönen, Shintaro Katayama, Liselotte Vesterlund, Eeva-Mari Jouhilahti, Mona Sheikhi, Elo Madissoon, Giuditta Filippini-Cattaneo, Marisa Jaconi, Anna Johnsson, Thomas R. Bürglin, Sten Linnarsson, Outi Hovatta and Juha Kere

The supplementary information contains followings;

Supplementary Figures

Supplementary Tables

Supplementary Notes

Supplementary References

SUPPLEMENTARY FIGURES

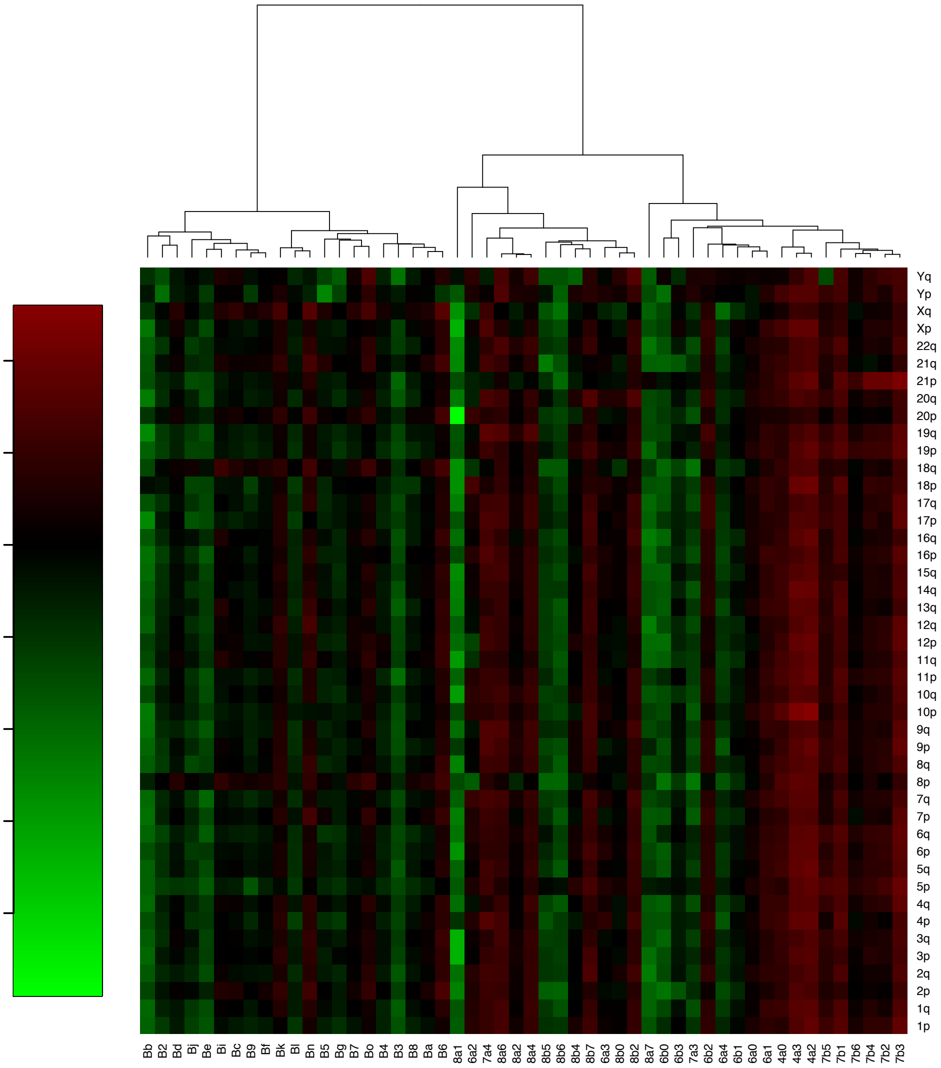

Supplementary Figure 1: **Overview of technical variation and biological variation.** This heatmap is comparison of $\log_{10}$ normalized expression levels with quantification and scaling by chromosome arms (y-axis) between samples in L146 libraries (x-axis); sample naming is as Supplementary table 1. Similarities between samples were compared by hierarchical clustering with Spearman correlation distance (in complete pair of observations) and Ward's linkage method. Variations between 50 pg of bulk human total RNAs (x-axis, beginning "B") represent only technical factors in equivalent RNA amount with day 3 blastomeres, while the other variations between blastomeres represent technical and biological factors. Because of scaling by chromosome arms, broad green/red contrast in blastomeres elucidated high variance of RNA content as in Figure 1d. In contrast, the technical replicates by bulk RNA we show relatively small variation, like bottom panel of Figure 1f.

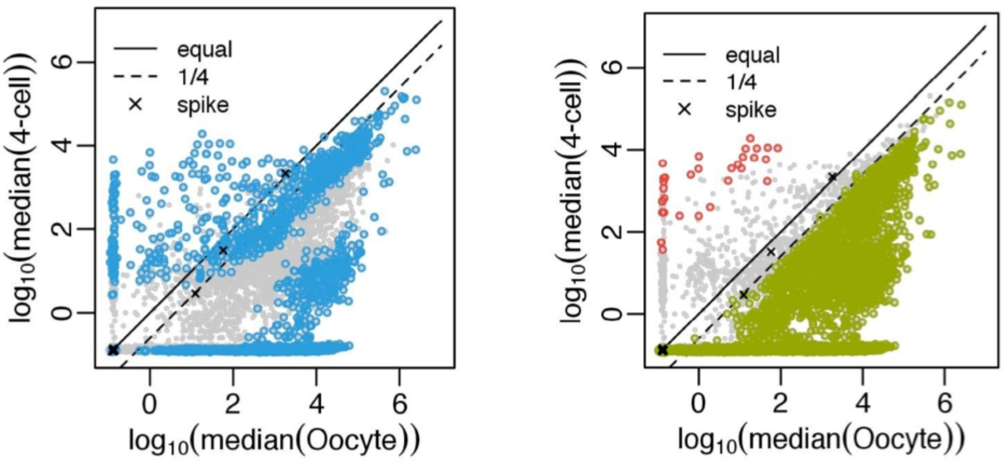

Normalization based on endogenous genes

Normalization based on synthetic RNA molecules ("spike-ins")

Supplementary Figure 2: **Comparison of normalization methods on sequenced oocytes and 4-cell stage blastomeres.** Both scatter plots are comparisons of expression levels between oocytes and 4-cell stage blastomeres in L185 with median representation of spike-in based normalized values; points in figures denote TFEs. Significantly regulated TFEs are colored; blue ones are by SAMseq[1], red are upregulated TFEs by SAMstrt[2], and green are downregulated or degraded TFEs by SAMstrt with "*in silico* dilution" compensating for cell cleavage effects (passive reduction by two cleavages; see Methods). Points 'x' are levels of spike-in RNAs. RPM (reads per million), which is the backbone of RPKM (reads per kilo-base per million) normalization in the previous studies[3,4] assumes "there are equivalent number of transcripts in all samples", and many other methods for RNA-seq[1] refer depth of all endogenous genes on similar assumption. However, such assumption does not fit the preimplantation development because of the cleavage divisions and the maternal transcript degradations; in consequence, for example, SAMseq results an overestimation of the upregulated/accumulated TFEs, and an underestimation of downregulated/degraded TFEs (left panel). Moreover, the spike-in RNAs, which we added equivalently into the samples (see Methods), were considered as upregulation. Therefore, we developed SAMstrt[2], which is an extension code for SAMseq enabling spike-in normalization and statistical testing based on "equivalent number of spike-ins in all samples". And it respects change of cellular RNA content, especially in the different stages of early development (right panel).

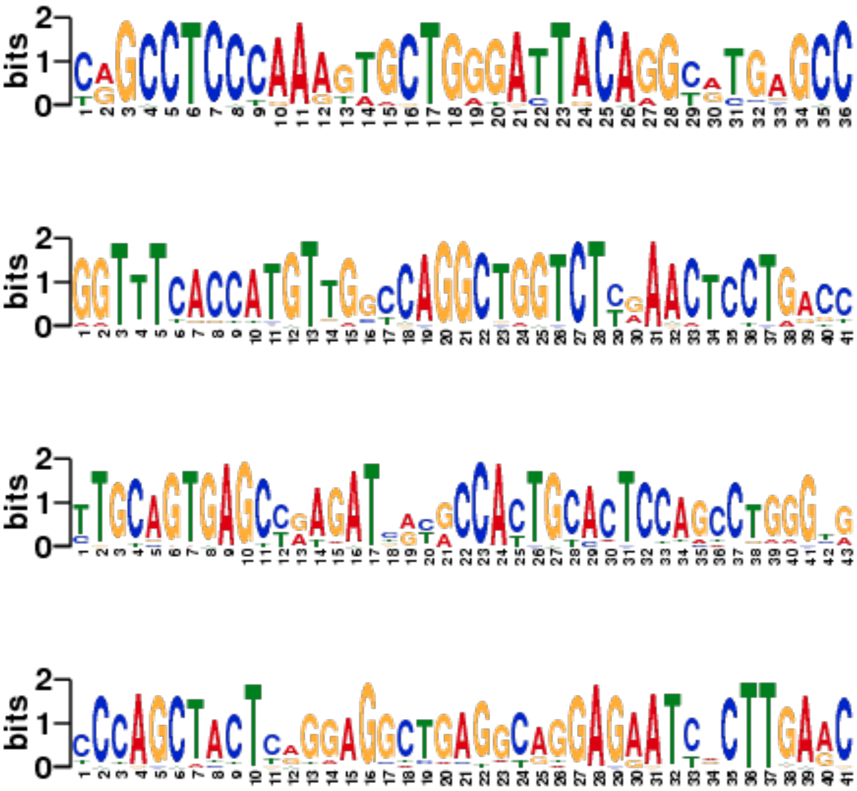

E=6.5×10^{-236}, 23 TFEs

E=2.6×10^{-253}, 23 TFEs

E=9.6×10^{-290}, 26 TFEs

E=6.1×10^{-219}, 22 TFEs

Supplementary Figure 3: Discovery of *de novo* genomic DNA motifs found associated with the 32 accumulated TFEs in the human early EGA. The motifs represented by position-specific probability matrices and the E-values representing significance of the motifs were estimated by MEME[5] (see also Supplementary Methods).

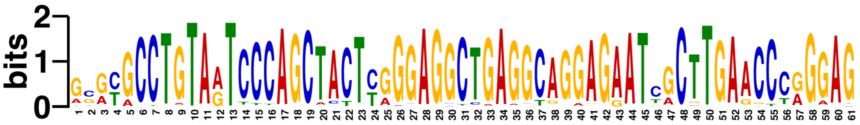

E=1.7×10^{-1775}, 89 TFEs

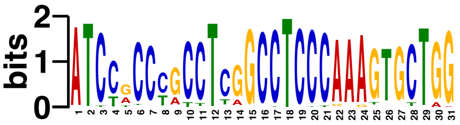

E=2.6×10^{-646}, 74 TFEs

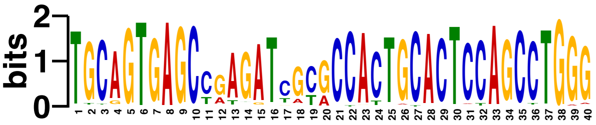

E=8.7×10^{-1043}, 82 TFEs

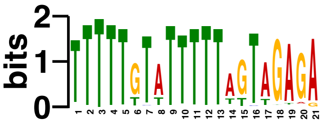

E=9.8×10^{-481}, 99 TFEs

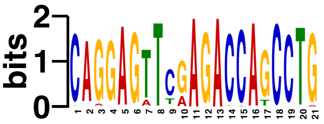

E=6.9×10^{-430}, 78 TFEs

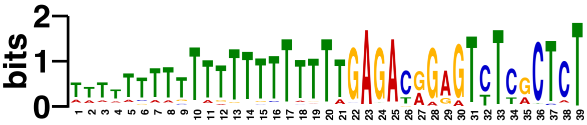

E=3.5×10^{-674}, 88 TFEs

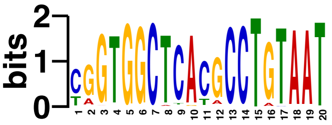

E=1.3×10^{-406}, 83 TFEs

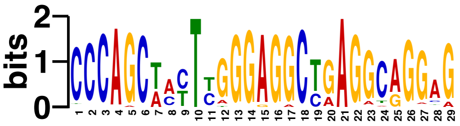

E=8.7×10^{-406}, 60 TFEs

Supplementary Figure 4: (*Continued on the next page.)*

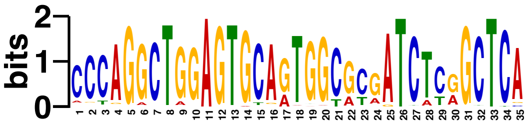

E=2.4×10^{-469}, 50 TFEs

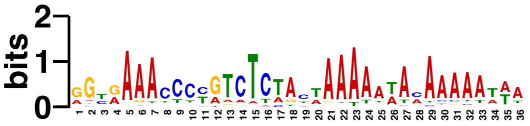

E=2.7×10^{-297}, 102 TFEs

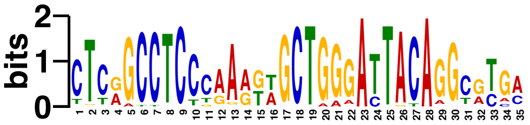

E=1.0×10^{-281}, 39 TFEs

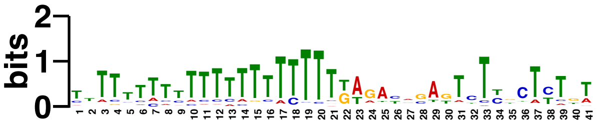

E=2.3×10^{-264}, 99 TFEs

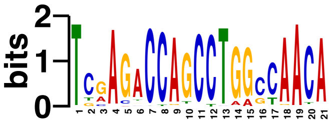

E=1.4×10^{-211}, 58 TFEs

Supplementary Figure 4: ***De novo*** **genomic DNA motifs around the 129 accumulated TFEs in the human late EGA.** *(Continued from the previous page)* The motifs represented by position-specific probability matrices and the E-values representing significance of the motifs were estimated by MEME[5] (see also Methods).

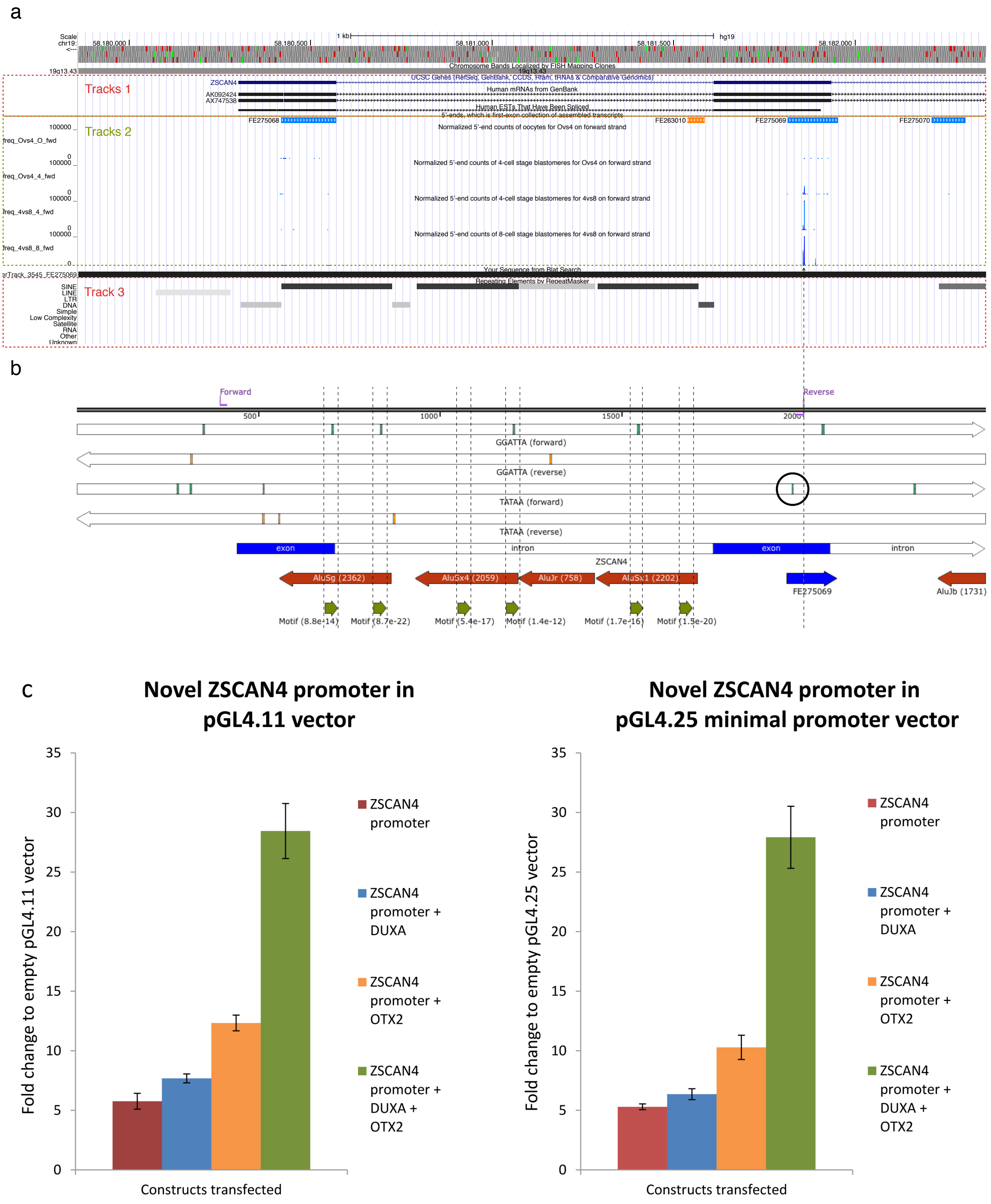

Supplementary Figure 5: *ZSCAN4* promoter construct and luciferase assay. (a) UCSC genome browser view of the *ZSCAN4* promoter region, -2,000 till +500 bp from the detected TFE peak FE275069. In Track 1 GenBank AK092424, (NM_152677), isolated from placenta and AX747578 (patent sequence from unknown source), are shown. Tracks 2 show the location of TFE peak FE275069 in the 2nd exon of known *ZSCAN4* transcript. Track 3 shows the five Alu elements identified in the *ZSCAN4* promoter region.

(b) Schematic illustration of Alu elements and *de novo* 36-bp motifs in the *ZSCAN4* promoter region. All five Alu elements are in the opposite orientation compared to *ZSCAN4* (orange arrows; number is Smith–Waterman score by RepeatMasker). All six 36-bp *de novo* motif similar sequences were overlapping with the three AluS elements (green arrows; number is similarity p-value by MEME), and four of the six had the GGATTA sequence, a known consensus sequence for PRD-like homeodomains (green bands in the top white arrow). Consensus TATAA sequence is located at -26 bp (encircled) from the FE275069 TFE peak (vertical dashed line to the right). Primers (Forward and Reverse in purple) were designed to obtain a 1605 bp amplicon containing the upstream region from the TFE FE275069 peak.

(c) Activity of the novel *ZSCAN4* 1605 bp promoter fragment. The 1605 bp fragment from the predicted *ZSCAN4* promoter was cloned into pGL4.11 and pGL4.25 luciferase reporter vectors and transfected into HEK293 cells. The presence of the *ZSCAN4* promoter fragment in pGL4.11 increased the luciferase expression 5-fold compared to empty pGL4.11 vector (left). With the cotransfection of *DUXA* and *OTX2* expression construct, alone or in combination, a further increase in fold induction was observed. The *ZSCAN4* promoter fragment vector pGL4.25 construct gave approximately the same results as the promoterless luciferase vector pGL4.11 (right). Thus the 1605 bp *ZSCAN4* promoter fragment is able to drive expression of downstream genes, and this *ZSCAN4* promoter driven expression is increased upon over-expression of PRD-domain containing transcription factors, such as *DUXA* and *OTX2*. Y-axis shows the fold change to empty vector +/- s.e.m (n=3 biological * 2 technical replicates).

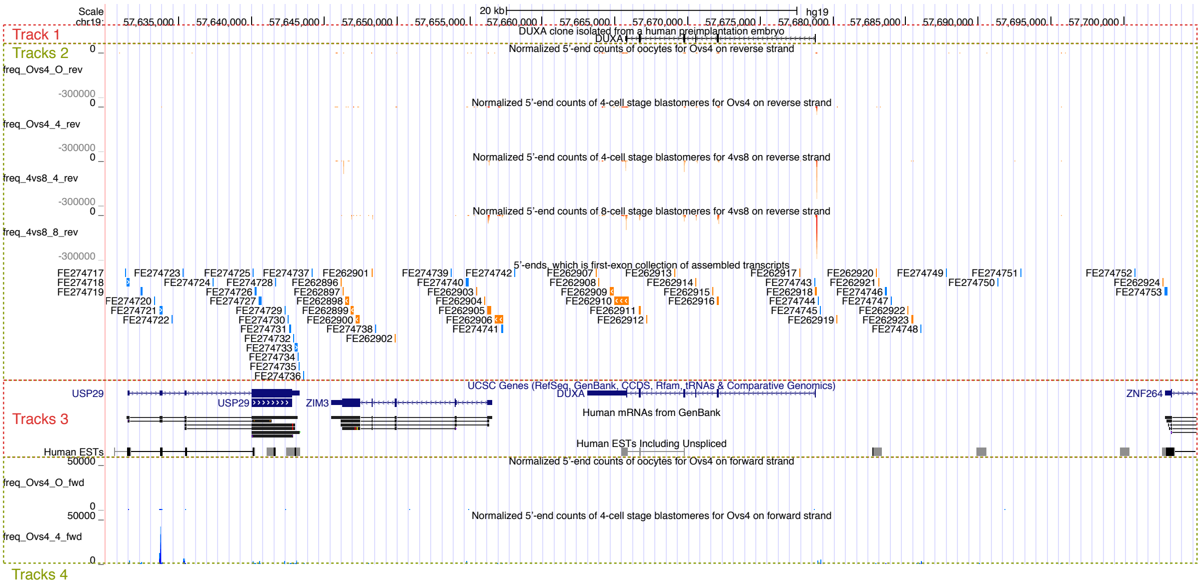

Supplementary Figure 6: Validation of *DUXA* transcript by cDNA cloning. Track 1 shows the alignment of the *DUXA* cDNA cloned from a human 8-cell stage embryo library synthesized using the method from Tang et al[1]. Tracks 2 visualize the expression peak in TFE, FE262918, which was significantly accumulated in both the oocyte to 4-cell and the 4-cell to 8-cell transitions (see also Figure 4b main paper). Tracks 3 show the previously predicted *DUXA* transcript which is located in the *PEG3*/*USP29* imprinted domain inbetween two Kruppel-type zinc-finger genes, *ZIM3* and *ZNF264*[6]. In order to validate the presence of a full-length *DUXA* transcript we designed primers based on the TFE peak FE262918 and connecting primers to the 3'UTR of predicted *DUXA* transcript downstream of this peak. Interestingly, we observed another peak, TFE FE274721 depicted in Track 4, accumulated during the same transitions and located at the second exon of imprinted neighborhood gene *USP29*. The colors in histograms (tracks 2 and 4) and TFEs reflect the strand specific STRT read alignments; blue refers to the forward strand (transcription from left to right), and orange to the reverse strand.

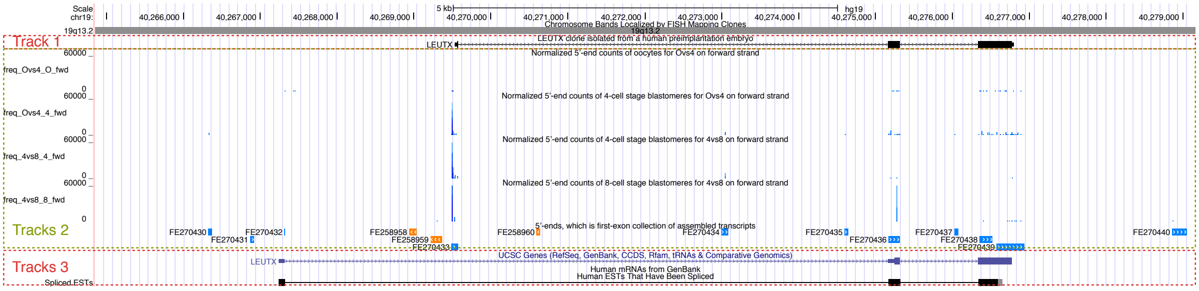

Supplementary Figure 7: Validation of *LEUTX* transcript by cDNA cloning. Track 1 shows the alignment of the *LEUTX* cDNA cloned from a human 8-cell stage embryo library. FE270433 was significantly accumulated in the oocyte to 4-cell stage transition (tracks 2 and Figure 4b), and is located in an intron of the predicted LEUTX transcript. Although there was no evidence of the predicted full-length form (tracks 3), we assumed the connection between the TFE and exon 3 of the predicted *LEUTX* for primer design, and isolated the cDNA clone from a human 8-cell stage embryo. Interestingly, the start codon in our novel exon provides a complete homeodomain whereas the homeodomain is truncated in the predicted *LEUTX*. The colors in histograms and TFEs (track 2) reflect the strand specific STRT read alignments; blue refers to the forward strand (transcription from left to right), and orange to the reverse strand.

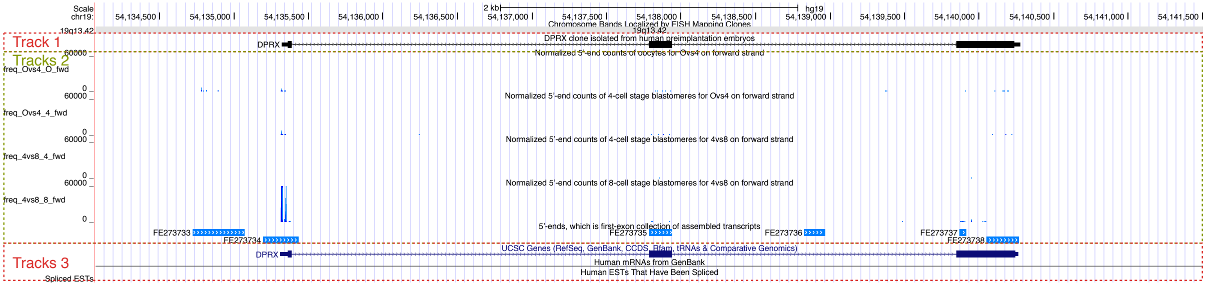

Supplementary Figure 8: Validation of *DPRX* transcript by cDNA cloning. Track 1 shows the alignment of the *DPRX* cDNA cloned from a human 8-cell stage embryo library. FE273734 was significantly accumulated in the 4-cell to 8-cell stage transition (tracks 2 and Figure 4e), and is located at the 5'-UTR of the predicted *DPRX* transcript. Although there was no evidence of the predicted full-length form (tracks 3), we used the predicted *DPRX* sequence for primer design, in order to isolate the cDNA clone from a human embryo. The blue color in histograms and TFEs (track 2) reflect the strand specific STRT read alignments to the forward strand (transcription from left to right).

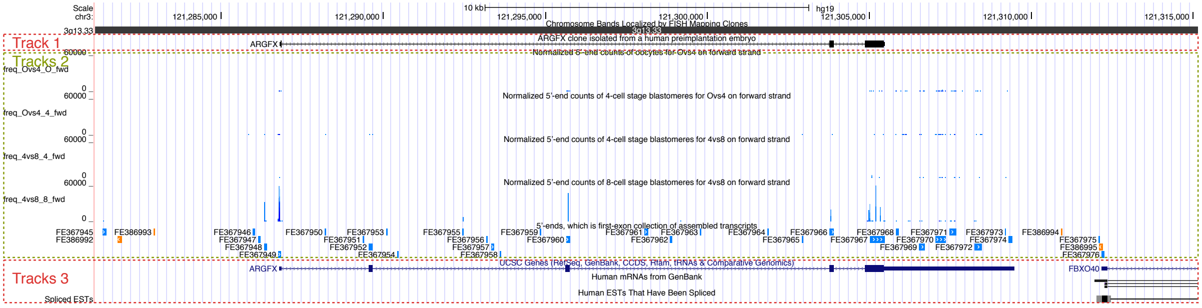

Supplementary Figure 9: Validation of *ARGFX* transcript by cDNA cloning. Track 1 shows the *ARGFX* expression in an 8-cell stage human embryo. FE367949 was significantly accumulated in the 4-cell to 8-cell stage transition (tracks 2 and Figure 4e), and is located at the 5'-UTR of predicted *ARGFX* transcript, although there was no evidence of the predicted full-length form (tracks 3). We used the predicted *ARGFX* sequence for primer design, and isolated the cDNA clone from a human embryo. Interestingly, the isolated transcript lacked exons 2 and 3 of the predicted form, however it did contain a complete homeobox. The first methionine in the first exon of the isolated *ARGFX* transcript is directly downstream of a kozak consensus sequence. The colors in histograms and TFEs (track 2) reflect the strand specific STRT read alignments; blue refers to the forward strand (transcription from left to right), and orange to the reverse strand.

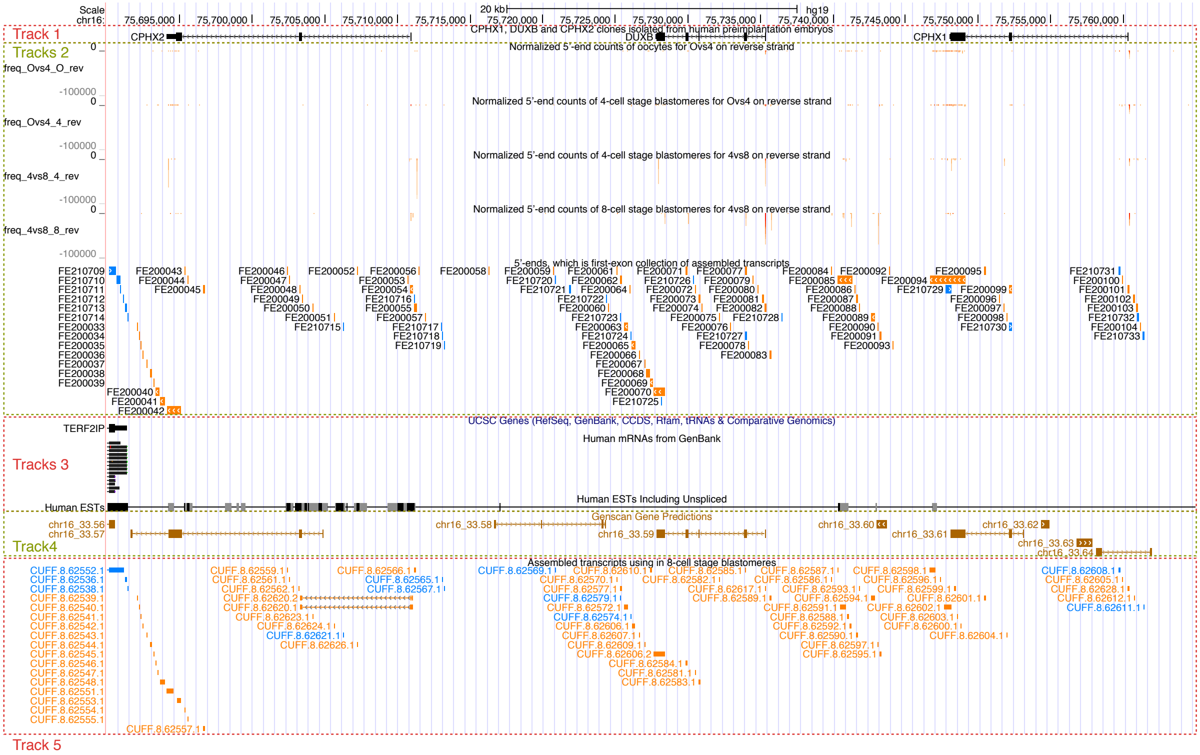

Supplementary Figure 10: Validation of *CPHX1*, *DUXB* and *CPHX2* transcripts by cDNA cloning. Track 1 shows the detected expression of *CPHX1*, *DUXB* and *CPHX2* genes in human 8-cell stage preimplantation embryos. Those genes are neighboring on the same strand within the chr16:75,690,001-75,765,000 region of the hg19 reference genome. These were maternal transcripts, and moreover the transcripts were accumulated in several cells in later developmental stages, as seen in Tracks 2 (see also Figure 4b main paper) showing our STRT read distribution and frequencies. Tracks 3 shows the lack of gene models and registered cDNAs, even though all of these genes were predicted. Track 4 shows predictions from various sources. Genscan gene prediction program suggested *DUXB* (chr16_33.59 of track 4) at the proximal downstream of FE200082. Also, the prediction (chr16_33.57 of track 4) and an assembled transcript of 8-cell stage STRT reads (CUFF.8.62620.1 of track 5) suggested the *CPHX2-like* gene at the proximal downstream of FE200054. Although we could find no evidence of a connection between the predicted *CPHX1-like* gene (chr16_33.61 at track 4) and the FE200101, we hypothesized that the FE200101 is in the first exon of *CPHX1*. We designed primer pairs for *CPHX1*, *DUXB* and *CPHX2* in order to amplify the putative complete ORFs from human 8-cell embryo. The resulting cDNA clones are shown in Track 1. The colors in histograms, TFEs and assembled transcripts (tracks 2 and 5) reflect the strand specific STRT read alignments; blue refers to the forward strand (transcription from left to right), and orange to the reverse strand.

Supplementary Table 1: Sample layout. These tables are statistics by sample types (rows) and STRT library IDs (columns). The former two tables are numbers of initially sampled eggs and embryos (a), and the actual numbers of cells (b). The latter two tables are number of qualified sampled eggs and embryos (c), and the actual numbers of cells (d).

(a)

**Stage**	**L146**	**L184**	**L185**	**L186**	**L187**	**L233**	**Total**
**Oocyte**	0	0	8	0	0	14	**22**
**Zygote**	0	0	0	0	32	30	**62**
**2-cell embryo**	0	0	0	0	0	4	**4**
**3-cell embryo**	0	0	0	0	0	1	**1**
**4-cell embryo**	1	1	8	3	0	2	**15**
**5-cell embryo**	0	1	0	0	0	0	**1**
**6-cell embryo**	2	1	0	1	0	0	**4**
**7-cell embryo**	2	0	4	2	0	0	**8**
**8-cell embryo**	2	8	0	4	0	0	**14**
**9-cell embryo**	0	0	2	2	0	0	**4**
**10-cell embryo**	0	0	1	1	0	0	**2**
**Total**	**7**	**11**	**23**	**13**	**32**	**51**	**137**

(b)

**Stage**	**L146**	**L184**	**L185**	**L186**	**L187**	**L233**	**Total**
**Oocyte**	0	0	8	0	0	14	**22**
**Zygote**	0	0	0	0	32	30	**62**
**2-cell blastomere**	0	0	0	0	0	5	**5**
**3-cell blastomere**	0	0	0	0	0	1	**1**
**4-cell blastomere**	4	4	29	11	0	6	**54**
**5-cell blastomere**	0	5	0	0	0	0	**5**
**6-cell blastomere**	9	5	0	5	0	0	**19**
**7-cell blastomere**	8	0	24	12	0	0	**44**
**8-cell blastomere**	14	46	0	26	0	0	**86**
**9-cell blastomere**	0	0	18	13	0	0	**31**
**10-cell blastomere**	0	0	9	10	0	0	**19**
**Total**	**35**	**60**	**88**	**77**	**32**	**56**	**348**

(c)

**Stage**	**L146**	**L184**	**L185**	**L186**	**L187**	**L233**	**Total**
**Oocyte**	0	0	6	0	0	14	**20**
**Zygote**	0	0	0	0	30	29	**59**
**2-cell embryo**	0	0	0	0	0	4	**4**
**3-cell embryo**	0	0	0	0	0	0	**0**
**4-cell embryo**	1	1	8	3	0	2	**15**
**5-cell embryo**	0	1	0	0	0	0	**1**
**6-cell embryo**	2	1	0	1	0	0	**4**
**7-cell embryo**	2	0	4	2	0	0	**8**
**8-cell embryo**	2	8	0	4	0	0	**14**
**9-cell embryo**	0	0	2	2	0	0	**4**
**10-cell embryo**	0	0	1	1	0	0	**2**
**Total**	**7**	**11**	**21**	**13**	**30**	**49**	**131**

(d)

**Stage**	**L146**	**L184**	**L185**	**L186**	**L187**	**L233**	**Total**
**Oocyte**	0	0	6	0	0	14	**20**
**Zygote**	0	0	0	0	30	29	**59**
**2-cell blastomere**	0	0	0	0	0	4	**4**
**3-cell blastomere**	0	0	0	0	0	0	**0**
**4-cell blastomere**	3	4	23	11	0	4	**45**
**5-cell blastomere**	0	5	0	0	0	0	**5**
**6-cell blastomere**	9	4	0	5	0	0	**18**
**7-cell blastomere**	8	0	22	8	0	0	**38**
**8-cell blastomere**	13	43	0	22	0	0	**78**
**9-cell blastomere**	0	0	17	11	0	0	**28**
**10-cell blastomere**	0	0	7	6	0	0	**13**
**Total**	**33**	**56**	**75**	**63**	**30**	**51**	**308**

SUPPLEMENTARY NOTES

Supplementary Note 1: Definition of transcript far 5'-ends (TFEs)

The STRT method used in this study gives the sequence of the 5'-end of polyA+ RNA and can be used for single cell sequencing. Alignment of STRT sequencing reads of single cells from early human preimplantation development revealed a complex transcriptome. Furthermore, the sequenced polyA+ RNA 5'end reads aligned to multiple locations even within single genes, or accumulated outside known CDS of annotated genes indicating novel promoters as for example within the human *PTP4A1* gene (Supplementary Figure SN1-1). Because the traditional quantitation of RNA expression by genes has insufficient resolution to explain such complex transcriptional regulation, we tried to identify a new high-resolution unit for quantitation of expression level by exons. We call this novel quantification unit as "transcript far 5'-ends (TFEs)".

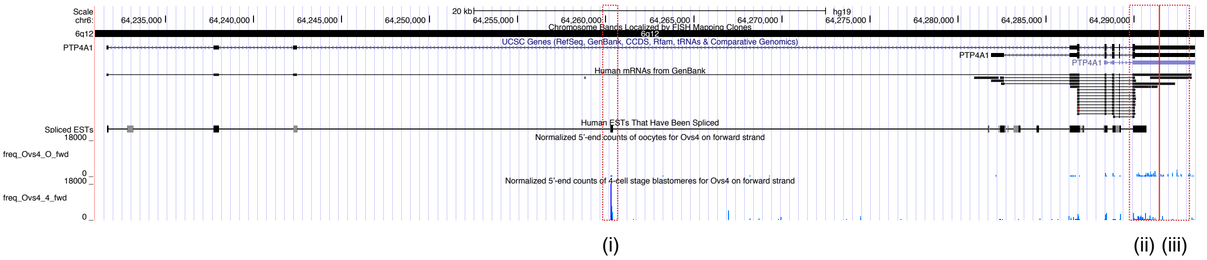

Supplementary Figure SN1-1: Visualization of the observed complex transcriptome properties. Here we visualize the alignment of STRT sequencing reads to the human *PTP4A1* gene in UCSC genome browser view. The top track shows the reference gene structures of *PTP4A1*, and the next two tracks visualize the transcripts from GenBank. The bottom two histograms are normalized STRT reads in oocytes (upper) and blastomeres of 4-cell stage embryo (lower). The height of the peak is proportional to the aligned reads. Regions (i) and (ii) point to the accumulated reads in the blastomeres whereas the region (iii) at the 3'-UTR shows a lower read count in the blastomeres than in oocytes. Interestingly, the region (i) is located at an intron of the reference *PTP4A1* gene structure proposing a novel promoter. TFEs are defined by merging the overlapping sequencing reads per region.

The alignments of five representative sample groups (oocyte, zygote, blastomeres isolated from day 2 and day 3 stage blastomeres, and 50 pg human brain total RNA) were assembled into putative transcript fragments (Supplementary Figure SN1-2a). We defined a total of 589,426 TFEs. There were relatively more TFEs found at intronic or unannotated genomic regions during human preimplantation development compared to the control sample group of human brain total RNA (Supplementary Figure SN1-2b), suggesting novel transcriptional start sites, novel genes, degrading transcripts, and/or transcription of repetitive elements.

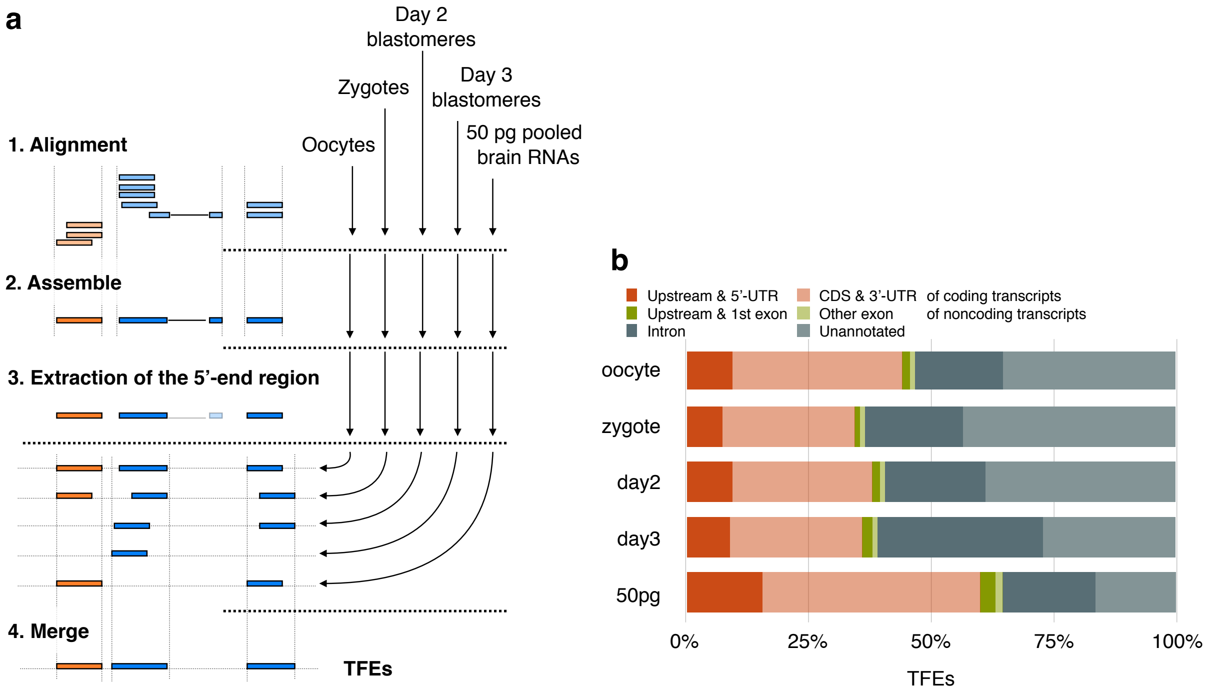

Supplementary Figure SN1-2: Explanation of the TFE definition and the distribution of TFE genome locations in human preimplantation development. (a) Schematic drawing explaining the bioinformatic approach to TFE definition. Step 1 to 3 that are performed in each representative sample group separately, include the alignment of the raw reads to the genome by TopHat, assembly of overlapping reads into potential transcripts by Cufflinks, and extraction of the first exon of the assembled transcripts as the far 5'-end exons of each transcript. The 4th and final step merges the overlapping extracted 5'-end exons from all sample groups in order to create a reference file where the merged fragments represent the TFEs as a collection of all potential first exons. Blue bars indicate the strand specific STRT read alignments on forward strand, and red bars on reverse strand. (b) Distribution of the genomic locations of the defined TFEs within the sample groups; four representative stages in human development (oocyte, zygote, day 2 and day 3 blastomeres) and one control sample group (50 pg pooled human brain total RNAs).

Supplementary Note 2: Consistency of STRT transcriptome with the sequencing data obtained by established sequencing methods

To confirm the consistency of the transcriptome observed in the current study with similar single-cell data performed using other RNA sequencing technologies, we first compared our TFEs to aligned traditional RNA-seq reads[3]. If the transcriptomes are similar, our TFEs should overlap with the traditional RNA-seq reads. The comparison is complicated by the fact that our method can only detect the 5'ends of polyA+ transcripts, whereas the traditional RNA-seq method covers the transcript regions broadly but with a more shallow coverage depth at

the 5'-end ends. However, our TFEs compiled from STRT reads were significantly supported by the published data in each developmental stage (Supplementary Table SN2-1, -2, -3 and -4; p-value<2.2×10^{-16} by Pearson's Chi-squared test with Yates' continuity correction). Second, we sequenced four single-zygote libraries using the sequencing method described by Tang *et al*[9] and compared the results with our STRT data, as well as with previous published results[3]. The STRT results were shown to be supported by our independent RNA-seq experiment (Supplementary Table SN2-5; p-value<2.2×10^{-16} by Pearson's Chi-squared test with Yates' continuity correction). Since the STRT method and older standard methods reveal similar transcriptomes in human preimplantation development we conclude that our TFEs are highly reliable quantification units.

Supplementary Table SN2-1: Overlap of oocyte specific TFEs detected by our STRT or RNA-seq by Yan, 2013. The table shows the number of TFEs that contain STRT reads derived from oocytes compared to the number of TFEs that contain traditional RNA-seq reads from Yan *et al*[3]. Oocytes from L233 library were used to specify the TFEs are expressed by oocytes in STRT data. The number in lower right corner points to the total number of TFEs.

		Our STRT data		
		Nr of TFEs expressed by oocytes	Nr of other defined TFEs	Total number of TFEs
**RNA-seq data by Yan et al.**	Nr of TFEs expressed by oocytes	110,297	140,737	**251,034**
	Nr of other defined TFEs	38,676	299,716	**338,392**
	Total number of TFEs	**148,973**	**440,453**	**589,426**
		(74.0% overlap)		

Supplementary Table SN2-2: Overlap of zygote specific TFEs detected by our STRT or RNA-seq by Yan, 2013. The table shows the number of TFEs that contain STRT reads derived from zygotes compared to the number of TFEs that contain traditional RNA-seq reads from Yan *et al*[3]. Zygotes from L233 library were used to specify the TFEs are expressed by zygotes in STRT data. The number in lower right corner points to the total number of TFEs defined over all developmental stages.

Our STRT data

		Nr of TFEs expressed by zygotes	Nr of other defined TFEs	Total number of TFEs
RNA-seq data by Yan et al.	Nr of TFEs expressed by zygotes	190,471	88,931	**279,402**
	Nr of other defined TFEs	95,328	214,696	**310,024**
	Total number of TFEs	**285,799**	**303,627**	**589,426**

(66.6% overlap)

Supplementary Table SN2-3: Overlap of 4-cell stage specific TFEs detected by our STRT or RNA-seq by Yan, 2013. The table shows the number of TFEs that contain STRT reads derived from 4-cell stage blastomeres compared to the number of TFEs that contain traditional RNA-seq reads from Yan *et al*[3]. 4-cell stage blastomeres from L185 library were used to specify the TFEs are expressed by 4-cell stage in STRT data. The number in lower right corner points to the total number of TFEs defined over all developmental stages.

		Our STRT data		
		Nr of TFEs expressed by 4-cell stage	Nr of other defined TFEs	Total number of TFEs
RNA-seq data by Yan et al.	Nr of TFEs expressed by 4-cell stage	54,630	241,777	**296,407**
	Nr of other defined TFEs	34,220	258,799	**293,019**
	Total number of TFEs	**88,850**	**500,576**	**589,426**

(61.5% overlap)

Supplementary Table SN2-4: Overlap of 8-cell stage specific TFEs detected by our STRT or RNA-seq by Yan, 2013. The table shows the number of TFEs that contain STRT reads derived from 8-cell stage blastomeres compared to the number of TFEs that contain traditional RNA-seq reads from Yan *et al*[3]. 8-cell stage blastomeres from L186 library were used to specify the TFEs are expressed by 8-cell stage in STRT data. The number in lower right corner points to the total number of TFEs defined over all developmental stages.

Our STRT data

		Nr of TFEs expressed by 8-cell stage	Nr of other defined TFEs	Total number of TFEs
**RNA-seq data by Yan et al.**	Nr of TFEs expressed by 8-cell stage	28,470	280,648	**309,118**
	Nr of other defined TFEs	9,809	270,499	**280,308**
	Total number of TFEs	**38,279**	**551,147**	**589,426**
		(74.4% overlap)		

Supplementary Table SN2-5: Overlap of zygote specific TFEs detected by our STRT or our independent RNA-seq experiment. The table shows the number of TFEs that contain STRT reads derived from zygotes compared to the number of TFEs that contain traditional RNA-seq reads from Yan *et al*[3]. Zygotes from L233 library were used to specify the TFEs are expressed by zygotes in STRT data. The number in lower right corner points to the total number of TFEs defined over all developmental stages.

		**Our STRT data**		
		Nr of TFEs expressed by zygotes	Nr of other defined TFEs	Total number of TFEs
**RNA-seq data from our independent experiment**	Nr of TFEs expressed by zygotes	212,817	165,743	**378,560**
	Nr of other defined TFEs	72,982	137,884	**210,866**
	Total number of TFEs	**285,799**	**303,627**	**589,426**
		(74.5% overlap)		

Supplementary Note 3: Extraction of expressed PRD-like homebox transcription factors (TFs)

At first we defined the loci of PRD-like homeobox TFs based on previously reports[7,10]. Among them, 18 TFs overlapped with 51 TFEs, which were significantly expressed in any oocyte, zygote or blastomere sample (q-value < 0.05 in comparison to zero reads by SAMstrt). 14 out of these 18 TFs overlapped with significantly expressed TFEs located at the 5'-UTR or proximal upstream regions.

SUPPLEMENTARY REFERENCES

1. Li, J. & Tibshirani, R. Finding consistent patterns: A nonparametric approach for identifying differential expression in RNA-Seq data. *Stat Methods Med Res* **22,** 519–536 (2013).
2. Katayama, S., Töhönen, V., Linnarsson, S. & Kere, J. SAMstrt: Statistical test for differential expression in single-cell transcriptome with spike-in normalization. *Bioinformatics* **29,** 2943–2945 (2013).
3. Yan, L. *et al.* Single-cell RNA-Seq profiling of human preimplantation embryos and embryonic stem cells. *Nat. Struct. Mol. Biol.* (2013). doi:10.1038/nsmb.2660
4. Xue, Z. *et al.* Genetic programs in human and mouse early embryos revealed by single-cell RNA sequencing. *Nature* (2013). doi:10.1038/nature12364
5. Bailey, T. L. & Elkan, C. Fitting a mixture model by expectation maximization to discover motifs in biopolymers. *Proc Int Conf Intell Syst Mol Biol* **2,** 28–36 (1994).
6. Kim, J., Bergmann, A., Wehri, E., Lu, X. & Stubbs, L. Imprinting and evolution of two Kruppel-type zinc-finger genes, ZIM3 and ZNF264, located in the PEG3/USP29 imprinted domain. *Genomics* **77,** 91–98 (2001).
7. Zhong, Y.-F. & Holland, P. W. H. The dynamics of vertebrate homeobox gene evolution: gain and loss of genes in mouse and human lineages. *BMC Evol. Biol.* **11,** 169 (2011).
8. Jolma, A. *et al.* DNA-Binding Specificities of Human Transcription Factors. *Cell* **152,** 327–339 (2013).
9. Tang, F. *et al.* RNA-Seq analysis to capture the transcriptome landscape of a single cell. *Nat Protoc* **5,** 516–535 (2010).
10. Holland, P. W. H., Booth, H. A. F. & Bruford, E. A. Classification and nomenclature of all human homeobox genes. *BMC Biol.* **5,** 47 (2007).
